# Aluminium Induced Endoplasmic Reticulum Stress Mediated Cell Death in SH-SY5Y Neuroblastoma Cell Line Is Independent of p53

**DOI:** 10.1371/journal.pone.0098409

**Published:** 2014-05-30

**Authors:** Syed Husain Mustafa Rizvi, Arshiya Parveen, Anoop K. Verma, Iqbal Ahmad, Md Arshad, Abbas Ali Mahdi

**Affiliations:** 1 Department of Biochemistry, King George’s Medical University, Lucknow, Uttar Pradesh, India; 2 Forensic Medicine & Toxicology, King George’s Medical University, Lucknow, Uttar Pradesh, India; 3 Fibre Toxicology Division, CSIR- Indian Institute of Toxicology Research, Lucknow, Uttar Pradesh, India; 4 Department of Zoology, Lucknow University, Lucknow, Uttar Pradesh, India; University of Colorado, Denver, United States of America

## Abstract

Aluminium (Al) is the third most abundant element in the earth’s crust and its compounds are used in the form of house hold utensils, medicines and in antiperspirant etc. Increasing number of evidences suggest the involvement of Al^+3^ ions in a variety of neurodegenerative disorders including Alzheimer’s disease. Here, we have attempted to investigate the role of Al in endoplasmic reticulum stress and the regulation of p53 during neuronal apoptosis using neuroblastoma cell line. We observed that Al caused oxidative stress by increasing ROS production and intracellular calcium levels together with depletion of intracellular GSH levels. We also studied modulation of key pro- and anti-apoptotic proteins and found significant alterations in the levels of Nrf2, NQO1, pAKT, p21, Bax, Bcl2, Aβ1-40 and Cyt c together with increase in endoplasmic reticulum (ER) stress related proteins like CHOP and caspase 12. However, with respect to the role of p53, we observed downregulation of its transcript as well as protein levels while analysis of its ubiquitination status revealed no significant changes. Not only did Al increase the activities of caspase 9, caspase 12 and caspase 3, but, by the use of peptide inhibitors of specific and pan-caspases, we observed significant protection against neuronal cell death upon inhibition of caspase 12, demonstrating the prominent role of endoplasmic reticulum stress generated responses in Al toxicity. Overall our findings suggest that Al induces ER stress and ROS generation which compromises the antioxidant defenses of neuronal cells thereby promoting neuronal apoptosis in p53 independent pathway.

## Introduction

The distinguishing features of neurodegenerative disorders include loss of neurons in the brain or spinal cord which, over a long period of time, result in the loss of a particular neuronal subtype or indiscriminate loss of neuronal populations. There have been reports that in Alzheimer’s disease and Huntington’s disease there is a loss of neurons [1], [2], while in Parkinson’s disease there is a specific and inadequate loss of dopaminergic neurons in the substantia nigra [3]. All these conditions disclose exclusive neuronal pathologies the precise mechanisms for neuronal loss are complex, making the identification of effective treatments indistinct.

Al which is the third most abundant element in the earth crust is not essential for organisms and no biological function has been assigned to it, however, its accumulation in tissues and organs has been reported to result in their dysfunction and toxicity [4]. Al compounds are being used in many industrial as well as house hold applications like water treatment, drugs and utensils, etc [5]. Though dietary intake of Al from food is small, the use of Al-containing antacids may provide doses of 50–1000 mg/day [6]. Studies have demonstrated that degenerating neurons in Alzheimer’s disease show high local Al concentrations [4], further its level in the brains of untreated DES patients have exceeded 25 µg/g in brain tissue [7]. Al can enter the central nervous system following systemic administration, causes behavioral impairments and neurolipofuscinogenesis [8].

Experimental evidences, both *in vitro* and *in vivo*, support that Al causes oxidative stress [9]–[11] though it is devoid of redox capacity in biological systems. Several lines of evidence suggest that apoptosis is the major mode of cell death induced by Al [12], [13]. A study by [14] indicated association of Al induced apoptosis with potential role of p53 signaling in Neuro 2a cells. Although mitochondria may play a central role in stress induced neuronal apoptosis by the activation of p53 [15], growing evidence now suggests that endoplasmic reticulum (ER) may also regulate neuronal apoptosis in stress conditions [16].

Perturbation of ER homeostasis affects protein folding and causes ER stress which is also implicated in many neurological disorders. ER stress can result in apoptosis [17] through activation of caspase-12. Expression of CHOP/Gadd153 is also associated with ER stress and recent micro-array studies revealed that CHOP/Gadd153 is one of highest inducible genes during ER stress [18].

Neurodegenerative disorders, like Alzheimer’s disease, exhibit characteristics of neuronal cell death however, their link with Al toxicity with respect to molecular mechanisms is still controversial. In the present study, we have attempted to explore the role of p53, if any, during oxidative and endoplasmic reticulum stress caused by Al(mal)_3_ (aluminium maltolate) in human neuroblastoma cell line. We have employed Al(mal)_3_ because of its electroneutral complex and provides significant amount of free aqueous Al at physiological pH and is soluble and stable from pH 3–10 [19]. We have demonstrated that Al(mal)_3_ induces apoptosis in neuroblastoma cells through ER stress pathway. Further, Al(mal)_3_ induced p53 dysregulation and it was not related to its post translational modification, but rather involves transcriptional disruption. This is the first report which shows that Al may induce p53-independent apoptosis in human neuron like cell line.

## Materials and Methods

### Materials

Aluminium chloride hexahydrate, Maltol, Tox 7 kit, (3-(4, 5-dimethyl- thiazol-2-yl)-2, 5-diphenyl tetrazolium bromide (MTT) dye and 2,7-dichlorofluorescein diacetate (DCFH-DA) were purchased from Sigma Chemical Co. Ltd. (St. Louis, MO, USA). DMEM/F12 cell culture medium, trypsin–EDTA, fetal bovine serum (FBS) and 100X antibiotic and antimycotic solution were purchased from (Gibco, USA). Caspase 12 and p53 antibodies were purchased from Abcam (Cambridge, UK), while Nrf2, NQO1, pAKT, p21, Bax and Bcl_2_ were procured from Santa Cruz (Texas, USA). CHOP/GADD153 and ubiquitin antibodies were purchased from BioLegend (USA) and Aβ1-40 was purchased from Millipore (MA, USA). Caspase 9, 3 and 12 activity kits as well as caspase 3, 9 and 12 inhibitors were purchased from BioVision Inc. (Mountain View, CA) while pan-caspase inhibitor (QVD-OPh) was procured from Abcam. All other chemicals were obtained from Merck unless otherwise mentioned.

### Cell Culture

SH-SY5Y human neuroblastoma cell line was purchased from National Centre for Cell Sciences (NCCS) Pune, India, and cultured in DMEM F-12 Hams (1∶1) supplemented with 10% fetal bovine serum, 0.2% sodium bicarbonate (Merck) and 1% antibiotic and antimycotic solution. Cultures were maintained at 37°C and 5% CO_2_ under high humid atmosphere. Medium was changed twice weekly and the cultures were split at a ratio of 1∶5 once a week.

### Preparation of Al(mal)_3_

Al(mal)_3_ was prepared from aluminium chloride hexahydrate and maltol (3-hydroxy-2-methyl-4H-pyran-4-one) according to the method described by Berthold *et al*. [20]. Briefly, for 10–15 g of complex, 15.5 g (122.8 mM) of maltol and 9.9 g (40.9 mM) of AlCl_3_.6H_2_O were dissolved in approximately 160 ml of deionized water accompanied by mild heating to facilitate dissolution. When most or all of the particulate material had dissolved, the pH was adjusted to 8.3. Subsequently, the mixture was heated to a temperature of 65°C that produced a finely divided precipitate, the formation of which was enhanced by stirring the solution. After cooling, the off-white crystals obtained were filtered, washed several times with acetone, and dried overnight in vacuum-dessicator. Yields of 75–85% of the theoretical 16.5 g of product were obtained.

### MTT Assay

MTT assay provides an indication of mitochondrial integrity and activity, which is interpreted as a measure of percent cell viability. MTT assay was done as per the method of Mosmann [21]. Briefly, cells were seeded in 96–well tissue culture plates (10,000 cells/well) in complete DMEM F-12 medium, followed by incubation in 5% CO_2_-95% atmosphere for 24 h at 37°C. Cells were exposed to different concentrations of Al(mal)_3_ (100 µM, 200 µM, 400 µM, 500 µM and 600 µM in a final volume of 100 µl media) followed by addition of MTT (10 µl per well of 5 mg/ml stock solution) 5 h prior to completion of incubation periods. Media was completely removed from each well and DMSO (200 µl) was added for solubilization of formazan crystals. After 10 min, absorbance was measured at 550 nm and relative percentage cell viability was calculated taking absorbance of control as 100 percent.

### LDH Activity-based Cytotoxicity Assay

Cells were seeded as described above for the MTT assay. The assay was performed as per the method used by Yang *et al.* [22] using TOX7 kit (Sigma) for estimation of live, apoptotic and necrotic populations. Briefly, after the treatment schedule, the floating apoptotic cells were collected from each well and medium was centrifuged at (240×g) at 4°C for 5 min. The LDH content from the pellets gave an index of apoptotic cell death (Ap) while that obtained from the medium gave an indication of necrotic population (Np). The enzymatic analysis from adherent cells was used as an index for live population (L_P_). For total LDH, 1/10 volume of LDH Assay Lysis Solution was added to separate set of wells and plate was returned to incubator for 45 min to release LDH from adherent as well as floating cells. Plate was centrifuged at 250×*g* for 5 min to pellet debris and aliquot transferred to clean flat-bottom plate. The enzymatic analysis of this supernatant aliquot gave total cellular LDH (T_P_). For interference from media, blank measurements were calculated by performing enzymatic analysis on cell-free media (Blk). The percentage of apoptotic and necrotic cell death was calculated as follows. % Live population = (L_P_-Blk/T_P_-Blk)*100, % Necrotic population = (N_P_- Blk/T_P_-Blk)*100, % Apoptotic population = (A_P_- Blk/T_P_-Blk)*100.

### Annexin V and PI Staining

Annexin V/PI staining was carried out using Annexin V/PI staining kit provided by BioLegend following manufacturer’s instructions, Briefly, cells were plated in 48 well tissue culture plate and treated with different concentration of Al(mal)_3_ for 24 h. Cells were then washed with PBS followed by incubation with prepared solution of annexin V/PI in annexin V binding buffer for 15 mins in dark at room temperature. Images were captured using Nikon eclipse Ti-S fluorescence microscope (Nikon Instruments Inc., Melville, NY) under 20X objective.

### Determination of Total ROS

Intracellular ROS generation was estimated by the method of Wan *et al.* [23] using 2′,7′-dichlorofluorescein diacetate (DCFH-DA) dye by measuring the conversion of non-fluorescent DCFH-DA to fluorescent dichlorofluorescein (DCF) within the cell using SYNERGY-HT multi-well reader (Bio-Tek, Winooski, USA). Briefly, cells seeded in black 96-well plate at a density of 10,000 cells/well were incubated with 10 µM DCFH-DA for 30 min at 37°C followed by incubation with desired treatments of Al(mal)_3_. The measurement of intracellular ROS was carried out during the course of the treatment period at 485 nm excitation and 535 nm emission wavelengths. This was further confirmed by fluorescence micrograph of cellular ROS. Briefly, cells were plated in 48-well tissue culture plate and treated with 10 µM DCFH-DA for 30 min followed by incubation with different concentrations of Al(mal)_3_ for one h at 37°C. Images were captured using Nikon eclipse Ti-S fluorescence microscope (Nikon Instruments Inc., Melville, NY) under 20X objective.

### Measurement of GSH Activity

Cells were treated with the fluorescent probe Cell Tracker Green CMFDA (Molecular Probes) solubilised in DMSO (Sigma) to 10 mM concentration and used at a final concentration of 1 µM. As in case of ROS measurement, cells were treated with CMFDA for 30 min in black 96-well tissue culture plate prior to treatment. Fluorescence quantitation was done at 517 nm with excitation at 492**nm. The level of reduced glutathione (GSH) in control cells was set to 1.

### Measurement of Intracellular Calcium

Calcium Green 1-AM dye was used at a final concentration of 5 µM to measure intracellular calcium levels and treatment of cells with dye and Al(mal)_3_ was carried out similar to ROS measurement, except that fluorescence was recorded at 531 nm with excitation at 506 nm. The level of intracellular calcium in control cells was set to 1.

### Real-time PCR

RNA was isolated from control and Al(mal)_3_ treated cells using RNAqueous kit (Ambion Inc., Austin, TX, USA) following the manufacturer’s protocol. RevertAid First Strand cDNA Synthesis kit (Fermentas, St. Leon-Rot, Germany) was used to synthesize cDNA from 100 ng of RNA. Real-time PCR was performed using Power SYBR green PCR master mix (Applied Biosystems, Foster City, CA, USA) on ABI 7500 Fast Real-Time PCR System (Applied Biosystems, Foster City, CA, USA) following the fast thermal cycling conditions: 95°C for 5 min and 40 cycles of 95°C for 15 s and 60°C for 1 min. The sequences of primers used are listed in Table 1. Expression levels were calculated relative to glyceraldehyde 3-phosphate dehydrogenase (GAPDH) as endogenous control.

**Table 1:** List of primer sequences used in real time PCR.

Bcl-2	F- 5′-GTGGAGGAGCTCTTCAGGGA-3′ R- 5′-AGGCACCCAGGGTGATGCAA-3′
Bax	F- 5′-GGCCCACCAGCTCTGAGCAGA-3′ R- 5′-GCCACGTGGGCGGTCCCAAAGT-3′
p53	F- 5′-CGCTGCCCCCACCATGAGC-3′ R- 5′-CTGGAGTCTTCCAGTGTGATGA-3′
CHOP	F- 5′-AGAACCAGGAAACGGAAACAGA-3′ R- 5′-TCTCCTTCATGCGCTGCTTT-3′
GAPDH	F- 5′-GTCAACGGATTTGGTCGTATTG-3′ R- 5′-TGGAGGGATCTCGCTCCTGGAAGAT-3′

### Isolation of Total Cellular Protein

After treatment, cells were pelleted, washed with ice-cold PBS and lysed in RIPA lysis buffer containing 150 mM NaCl, 1% NP-40, 0.25% SDS, 1 mM EDTA, 1 mM PMSF and 1 mM sodium orthovanadate in 50 mM Tris-Cl (pH 7.4). Protease inhibitor cocktail was added fresh prior to lysis. Following incubation in lysis buffer for 1 h, the lysate was gently vortexed for 15 sec and supernatant was collected by centrifugation at 17,000×g for 15 min and stored in aliquots at −80°C. Protein content was quantified using Lowry’s method. To prepare cytoplasmic fractions, the method established by Janssen and Sen [24] was followed.

### Immunoblot Analysis

Forty to sixty microgram of protein sample was separated on 10% SDS– polyacrylamide gel electrophoresis and electro-blotted on PVDF membrane. The membrane was incubated for 2 h with specific polyclonal IgG antibodies of Nrf2, NQO1, pAKT, p21, Bax, Bcl_2_, Cyt c, caspase-12, p53, Gadd153/CHOP, ubiquitin or Aβ1-40. After 30 min washing with TBS-T, respective HRP-conjugated secondary antibodies were added for 1 h at room temperature. Immunoblot was revealed using Immobilon Western Chemiluminescent HRP substrate kit (Millipore Corporation, MA, USA). *β*-actin was used as internal standard. PageRuler Prestained Protein Ladder (5 µl) (Thermo, EU) was used to determine molecular weight of the protein bands. Densitometry of the bands obtained was done using NIH software Image J version 1.41 (USA). Band areas were calculated by densitometric scanning and result expressed as Arbitrary Units for each experimental band.

### Immunoprecipitation and Immunoblotting to Detect p53 Ubiquitination

Ubiquitination experiment was done by the method of Bloom and Pagano [25] with slight modifications. Briefly, 4 h prior to the completion of exposure time, cells were treated with 5 µM proteasome inhibitor MG132 (Abcam). Cells were lysed in protein extraction buffer containing 2 mM NEM to block the activity of isopeptidases and deubiquitylating enzymes. Cell lysate containing 500 µg protein was incubated overnight at 4°C with anti-ubiquitin antibody followed by incubation with 25 µl Protein-A/G PLUS agarose beads for 7 h. The lysate-beads mixture was centrifuged at 10,000×g and after rinsing thrice with lysis buffer, the pelleted beads were boiled with 2× SDS loading dye before running in a 10% SDS-PAGE. The proteins were thereafter transferred onto a PVDF membrane and probed with anti-p53 antibody. Lysates from a p53-null cell line HL60 were used as negative control.

### Caspases Activity

Cells seeded in 75 cm^2^ flask were treated with Al(mal)_3_ and cytosolic lysates were extracted and enzymatic activities of caspase-3, -9 and -12 were measured using Caspase-3 Colorimetric Assay Kit, Caspase-9 Colorimetric Assay Kit and Caspase-12 Fluorometric Assay Kit respectively (BioVision) according to manufacturer’s instructions. The absorbance was measured at 405 nm for caspase-3 and caspase-9, while for caspase-12 fluorescence was recorded at 505 nm emission with 400 nm excitation filter in a SYNERGY-HT multi-well plate reader (Bio-Tek).

### Caspases Inhibition

After 24 h of plating, cells were treated with caspase inhibitors namely pan-caspase inhibitor (QVD-OPh), caspase-3 inhibitor (Z-DEVD-FMK), caspase-9 inhibitor (Z-LEHD-FMK) and caspase-12 inhibitor (Z-ATAD-FMK) along with Al(mal)_3._ At the end of incubation, MTT and LDH assays were performed to assess the cell viability and apoptosis inhibition. The concentration of the respective inhibitors was used as per the manufacturer’s protocol.

### Statistical Analysis

All experiments were performed for a minimum of three times and results have been presented as mean ± SE. Statistical analysis was performed using SPSS 14.0 statistical package (SPSS Inc., Chicago, IL, USA). Statistical significance of the results was determined using one-way ANOVA by Tukey’s multiple comparison test. Differences were considered statistically significant at P<0.05.

## Results

### Al(mal)_3_ Induces Morphological Changes in SH-SY5Y Cells

**Figure 1:**
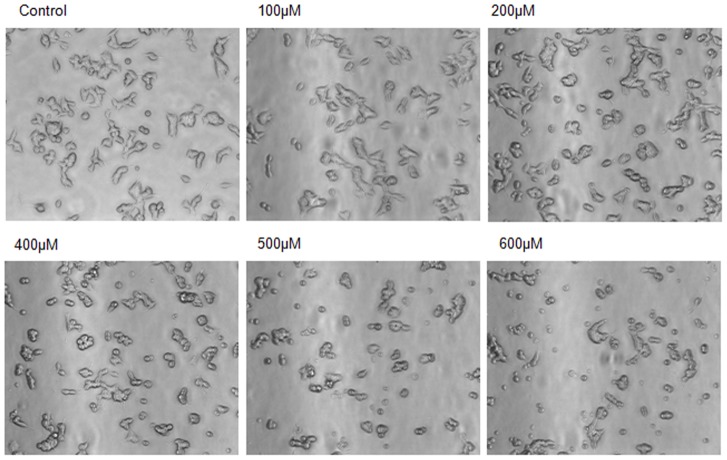
Al(mal)_3_ induced morphological changes in neuroblastoma cells. Phase-contrast image (20X objective) of the monolayers showing Al(mal)_3_ induced cell loss and morphological changes in neuroblastoma cells at different concentration of Al(mal)_3_ in cell culture plates.

We exposed neuroblastoma cells to different concentrations of Al(mal)_3_ (100 µM to 600 µM) for 24 h in 6-well tissue culture plates. The cell morphology was assessed using phase contrast microscopy under 20X objective (Figure 1) which clearly revealed cell loss from the monolayer as well as shrinkage and deformation of cell bodies at 400 µM and higher concentrations of Al(mal)_3_.

### Al(mal)_3_ Causes Both Apoptotic and Necrotic Cell Death

**Figure 2:**
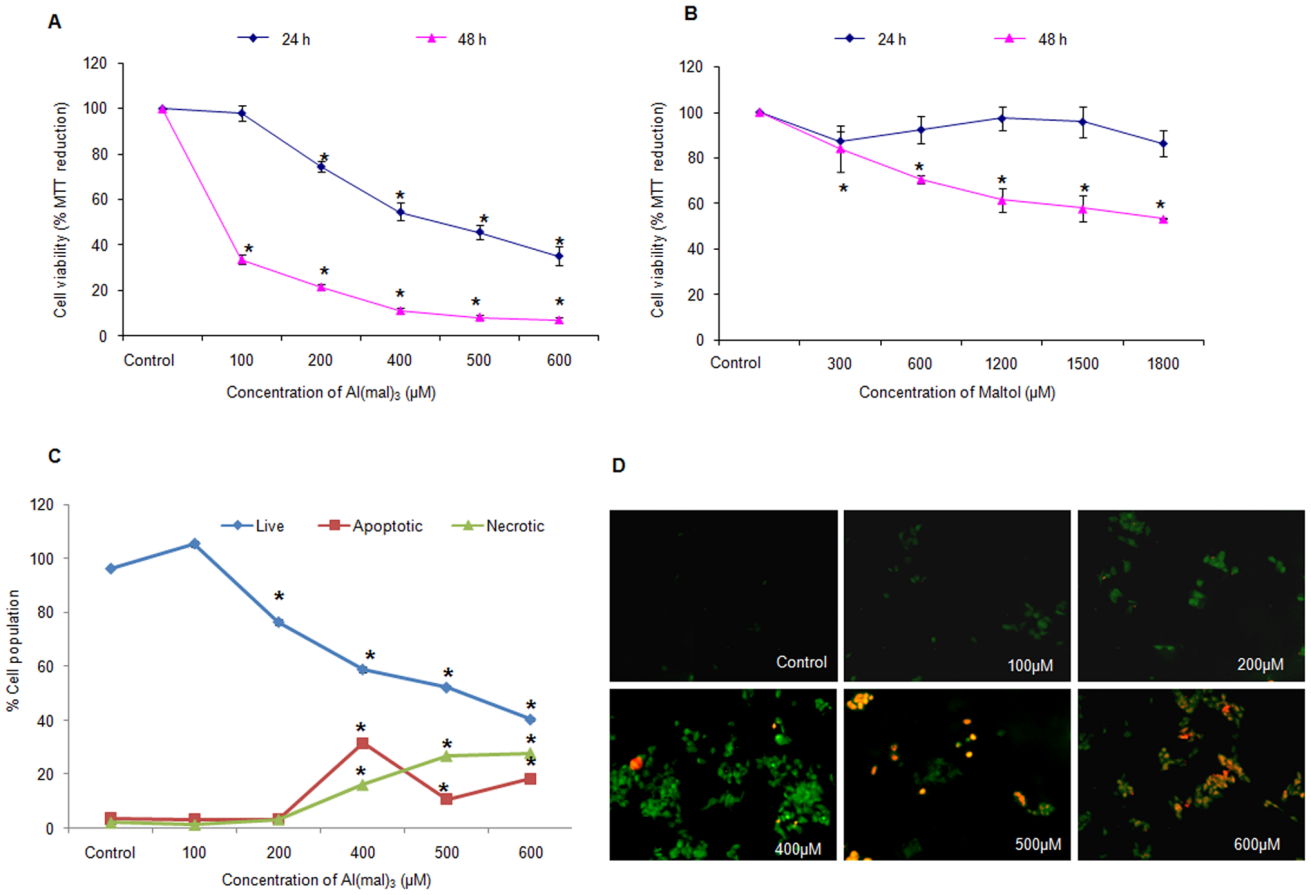
Al(mal)_3_ induced both apoptotic and necrotic cell death. Viability of neuroblastoma cells (10,000 cells/well) was evaluated by MTT assay in 96-well plates at 24 h and 48 h exposure to (A) concentrations of Al(mal)_3_ varying between 100 µM–600 µM and (B) Maltol alone at 3x concentration corresponding to each of Al(mal)_3_ concentration. (C) Live, Apoptotic and necrotic populations estimated using LDH activity-based cytotoxicity assay and (D) Fluorescence microscopy of Annexin V/PI staining of neuroblastoma cells 24 h post Al(mal)_3_ exposure (100 µM–600 µM) under 20X objective. The data are represented as means ±SE of three independent experiments. *P<0.05 vs. control.

We exposed neuroblastoma cells to different concentrations of Al(mal)_3_ (100 µM to 600 µM) for 24 h and 48 h. A 3x concentration of maltol alone was also included as control for each concentration of Al(mal)_3_. MTT assay results showed that at 100 µM and 200 µM concentrations, cell viability reduced to 98.3% and 74.5% respectively at 24 h which further dropped to 33.58% and 21.51% at 48 h (Figure 2A). Thus, significant cell death could be observed at 400 µM, 500 µM and 600 µM concentrations of Al(mal)_3_ at 24 h. At a concentration of 400 µM, only 54.6% cells were viable at 24 h and no significant cell death was observed upon treatment with its respective maltol control (1200 µM maltol; Figure 2B), hence, we selected 24 h time period for further experiments.

The percentage of live, apoptotic and necrotic populations estimated using Lactate dehydrogenase (LDH) assay revealed that significant apoptotic and necrotic cell death occurred upon exposure to 400 µM, 500 µM and 600 µM concentration of Al(mal)_3_ (Figure 2C). At 400 µM concentration, out of the approximate 50% population undergoing cell death, 31.6% cells were apoptotic as against 16.19% necrotic population. However, at higher concentrations of Al(mal)_3_, that is at 500 µM and 600 µM, necrosis was found to be predominant. This was further evaluated by fluorescence microscopy of annexinV/PI staining which confirmed that at 500 µM and 600 µM concentrations Al(mal)_3_ induced necrosis, while, at 400 µM, significant apoptosis was being induced (Figure 2D).

### Al(mal)_3_ Induces Oxidative Stress and Disturbs the Antioxidant Defenses within Neuroblastoma Cells

**Figure 3:**
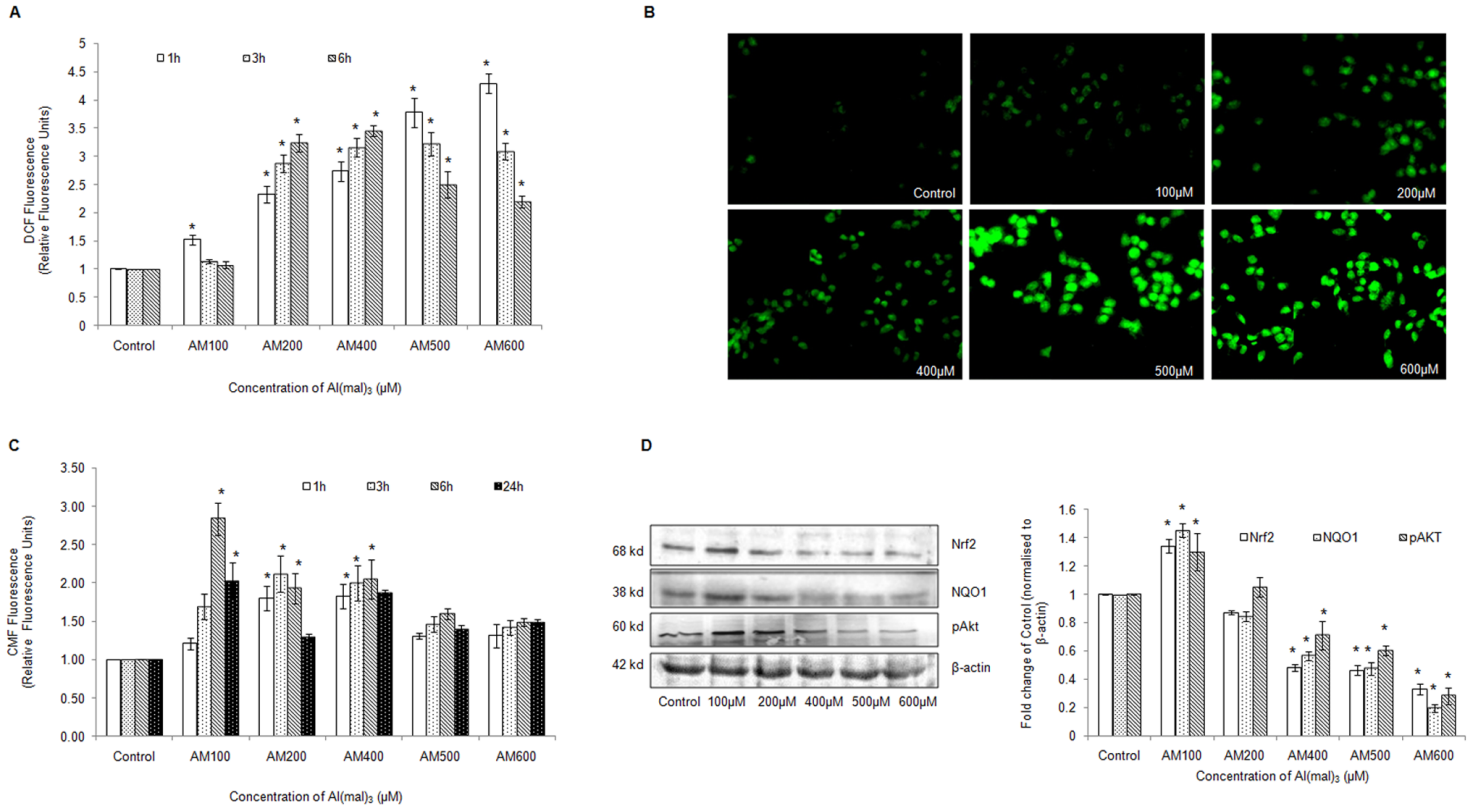
Al(mal)_3_ compromised antioxidant defenses of neuroblastoma cells. (A) ROS generation was assessed in terms of relative fluorescence units using 10 µM DCFH-DA in neuroblastoma cells after 1 h, 3 h and 6 h exposure to Al(mal)_3_ in black-bottomed 96-well plates. (B) Fluorescence micrographs of ROS generation at (100 µM–600 µM) Al(mal)_3_ concentrations in neuroblastoma cells obtained at 20X objective after 1 h treatment. (C) GSH levels were assessed in terms of relative fluorescence units using 5 µM CMFDA dye at 1 h, 3 h, 6 h and 24 h of Al(mal)_3_ exposure. After conjugation with GSH, CMFDA is hydrolyzed to the fluorescent 5-chloromethyl-fluorescein (CMF) by cellular esterases. (D) Western blot analysis of Nrf2, NQO1 and pAKT at 24 h of Al(mal)_3_ treatment (100 µM–600 µM). Band intensities were calculated by densitometry and change in protein expression (Al-treated) was calculated with respect to controls and expressed as fold change in graph. Results were normalized to *β*-actin. The data are represented as means ±SE of three independent experiments. *P<0.05 vs. control.

ROS generation was assessed at 1 h, 3 h and 6 h after treatment with Al(mal)_3_ (100 µM to 600 µM) concentrations. Significant elevation in ROS levels could be observed at all the tested time intervals and doses, however, maximum ROS levels were detected at 500 µM and 600 µM during the first hour (Figure 3A). Fluorescence micrographs of DCFH-DA stained cells further confirmed the above fluorometric findings (Figure 3B). GSH levels estimated at 1 h, 3 h, 6 h and 24 h after treatment with Al(mal)_3_ revealed maximum levels at 100 µM, 200 µM and 400 µM Al(mal)_3_ at 3 and 6 h (Figure 3C).

We next assessed protein expression of oxidative stress and cell survival related proteins by western blotting and found that except at 100 µM concentration, the levels of Nrf2 and its downstream molecule NQO1 were significantly suppressed at higher Al(mal)_3_ concentrations. p-AKT (phosphorylated Akt), which is a cell survival protein, also showed a similar expression pattern (Figure 3D). The data suggests Al evokes apoptosis of SH-SY5Y cells by disruption of survival pathways, and this disruption involves a free radical component.

### Al(mal)_3_ Induces Expression of Apoptosis Related Proteins

**Figure 4:**
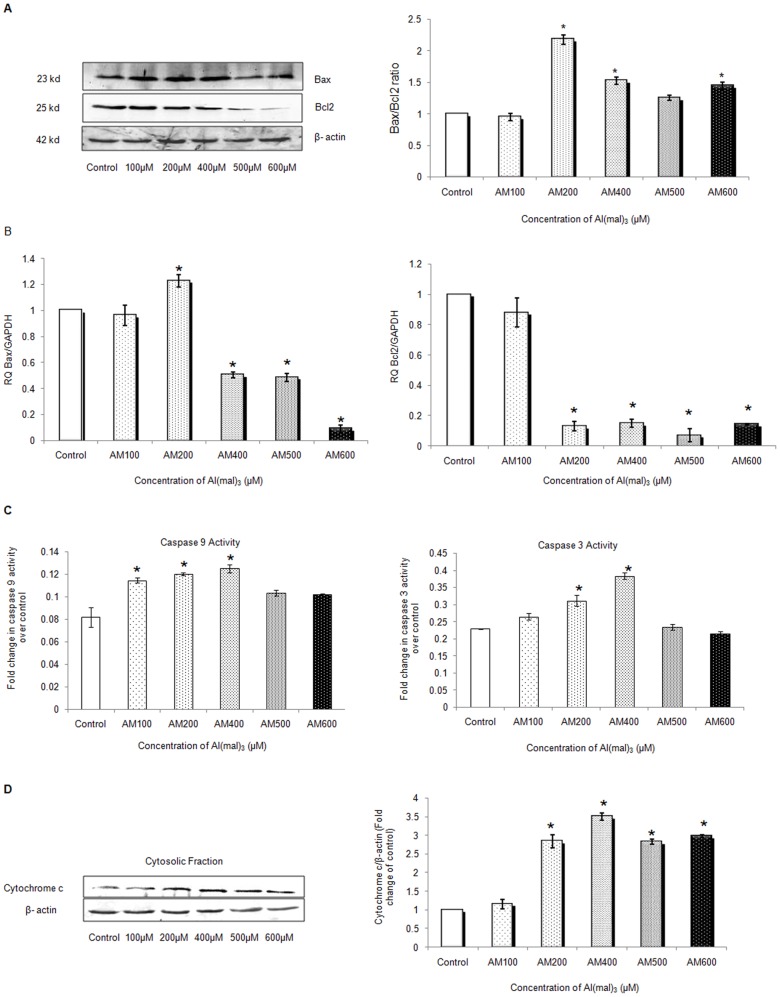
Al(mal)_3_ altered the levels of key apoptotic markers. (A) Ratio of Bax and Bcl_2_ was determined using western blot analysis of protein obtained after 24 h exposure to Al(mal)_3_ (100 µM–600 µM). Band intensities were calculated by densitometry and change in protein expression (Al-treated) was calculated with respect to controls and expressed as fold change in graph. Results were normalized to *β*-actin and then the ratio was obtained. (B) Relative quantification (RQ) of mRNA expression of Bax and Bcl_2_ were determined by real time PCR, endogenous control used was GAPDH. (C) Caspase 9 and Caspase 3 activity was assessed in cell lysates of the SH-SY5Y following treatment with Al(mal)_3_ (100 µM–600 µM) by spectrophotometric detection of the chromophore p-nitroaniline (pNA) formed after cleavage from the labeled substrate LEHD-pNA and DEVD-pNA using microtiter plate reader at 400 nm or 405 nm. (D) Western blot analysis of cytochrome c (Cyt c) in cytosolic fraction of Al(mal)_3_ treated SH-SY5Y neuroblastoma cells at 24 h, results were normalized to *β*-actin. The data are represented as means ±SE of three independent experiments. *P<0.05 vs. control.

We assessed protein and mRNA expression of apoptosis related proteins by western blotting and real time PCR. Bax, a pro-apoptotic protein significantly increased at 200 µM concentration (Figure 4A) and its mRNA expression also showed a similar pattern (Figure 4B). Bcl_2_, which is an anti-apoptotic protein, was down regulated at all the concentrations of Al(mal)_3_ (Figure 4A), though slight upregulation of its mRNA could be observed at 100 µM Al(mal)_3_ concentration (Figure 4B).

A significant increase in caspase 9 enzymatic activity was observed at 100 µM, 200 µM and 400 µM concentration of Al(mal)_3_ with respect to control (Figure 4C), while that of caspase-3 was observed at 200 µM and 400 µM concentration (Figure 4C). Western blot analysis of cytochrome c (Cyt c), an intermediate in the apoptotic process, revealed considerable increase at all the concentrations of Al(mal)_3_ (Figure 4D). The data further confirms that at lower concentrations, Al(mal)_3_ induces apoptotic cell death in neuroblastoma cell line.

### Al(mal)_3_ Perturbs the Calcium Fluxes and Induces ER Stress within the Neuroblastoma Cells

**Figure 5:**
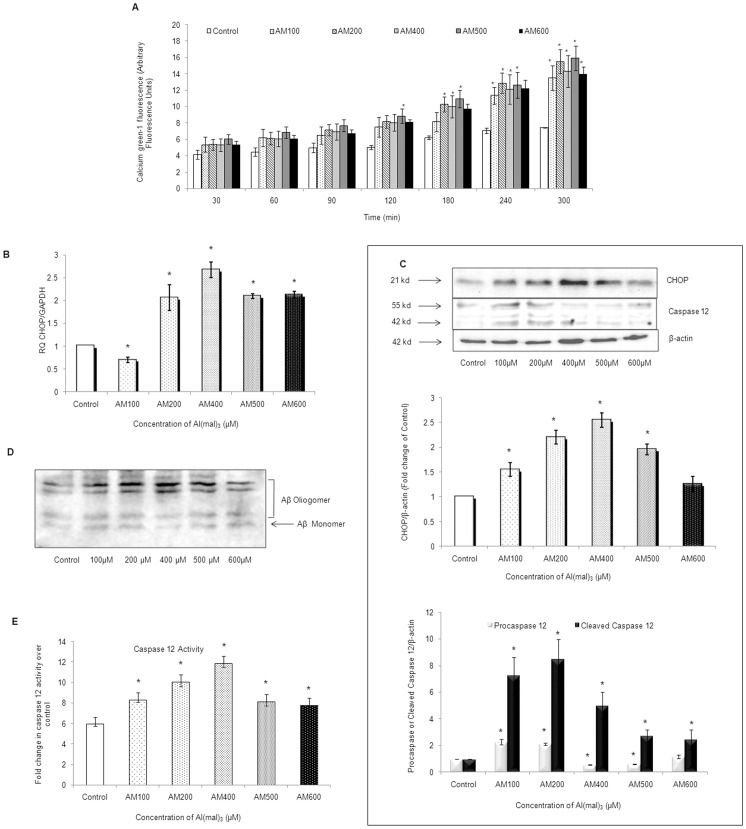
Al(mal)_3_ induced ER stress in neuroblastoma cells. (A) Intracellular calcium levels were determined in terms of arbitrary fluorescence units using 5 µM calcium green-1 AM dye at time periods ranging from 30 mins to 300 mins of Al(mal)_3_ exposure (100 µM–600 µM). Upon binding calcium ions, an increase in fluorescence of the respective dye is observed. (B) Relative quantification (RQ) of CHOP mRNA was done by Real time PCR following Al(mal)_3_ treatment (100 µM–600 µM) for 24 h. GAPDH was used as endogenous control. (C) Western blot analysis of CHOP and caspase 12 at 24 h of Al(mal)_3_ treatment (100 µM–600 µM). Band intensities were calculated by densitometry and change in protein expression (Al-treated) was calculated with respect to controls and expressed as fold change in graph. Results were normalized to *β*-actin. (D) Western blot analysis of Aβ(1–40) following 24 h of Al(mal)_3_ treatment (100 µM–600 µM) showed oligomers formation at different concentrations of Al(mal)_3_. (E) Enzymatic activity of caspase 12 was assessed in cell lysates of the SH-SY5Y following treatment with Al(mal)_3_ (100 µM–600 µM) by the detection of cleavage of substrate ATAD-AFC using fluorimeter (Ex/Em 400/505). The data are represented as means ±SE of three independent experiments.*P<0.05 vs. control;^ #^P<0.05 vs. Al(mal)_3_ treatment.

Perturbation of intracellular calcium levels is implicated in ER stress which was estimated using calcium green-1 AM dye at various time intervals ranging from 30 min to 300 min after exposure to Al(mal)_3_. Fluorometric estimation revealed that cytosolic calcium increased as the Al(mal)_3_ concentration increased and maximum levels were recorded for 200 µM at all the tested time periods (Figure 5A).

ER stress related protein CHOP/GADD153 is highly expressed during ER stress. Its m-RNA was upregulated at all the concentrations of Al(mal)_3_ except 100 µM (Figure 5B). Besides this, its protein expression pattern revealed significant increase at all the concentrations of Al(mal)_3_ and maximum levels were observed at 400 µM concentration (Figure 5C). While significant cleavage of pro-caspases 12 into active cleaved bands was observed at 200 µM and 400 µM, its enzymatic activity was also significantly increased (Figure 5C and Figure 5E). ER is also the site of amyloid beta (Aβ) generation. Western blot analysis revealed that Al(mal)_3_ treatment also enhanced the protein expression as well as the oligomeric forms of Aβ1-40 (Figure 5E). The data suggests that Al(mal)_3_ induced neuroblastoma insufficiencies involve dysregulation in ER.

### Al(mal)_3_ Induces Apoptosis in p53-independent Manner

**Figure 6:**
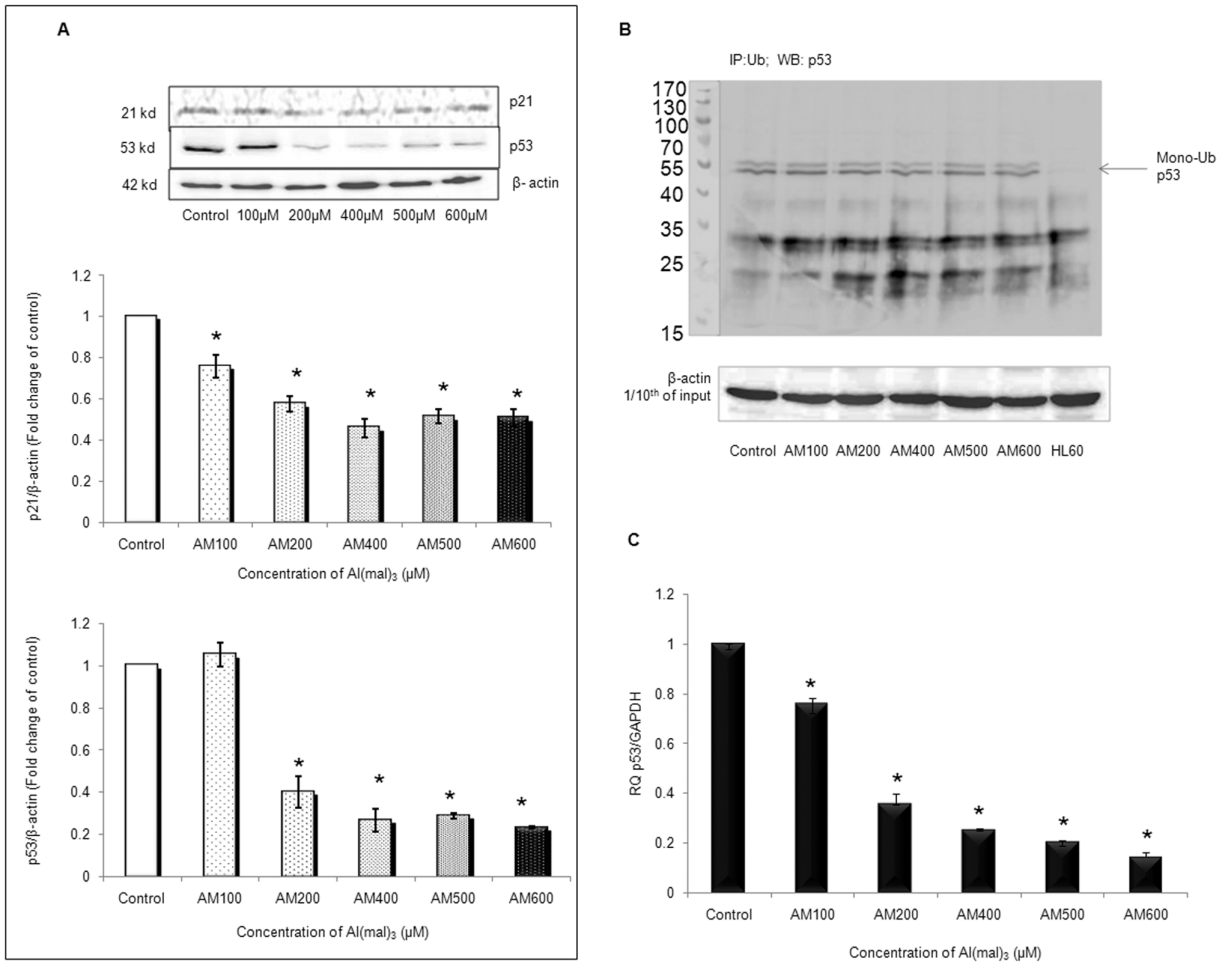
Al(mal)_3_ induced apoptosis in p53 independent manner. (A) Western blot analysis of p53 and p21 at 24 h of Al(mal)_3_ treatment (100 µM–600 µM). Band intensities were calculated by densitometry and change in protein expression (Al(mal)_3_ treated) was calculated with respect to controls and expressed as fold change in graph. Results were normalized to *β*-actin. (B) Status of p53 ubiquitination at different concentrations of Al(mal)_3_ (100 µM–600 µM) at 24 h. Total cell lysates were immunoprecipitated with anti-ubiquitin antibody followed by immunoblotting with anti-p53 antibody, p53-null cell line HL60 were used as negative control. (C) Relative quantification (RQ) of p53 mRNA expression was determined by real time PCR following Al(mal)_3_ treatment (100 µM–600 µM) at 24 h. GAPDH was used as endogenous control. The data are represented as means ±SE of three independent experiments. *P<0.05 vs. control.

In order to further delineate the mechanism implicated in Al induced neurotoxicity, we planned to explore the involvement of p53 and its associated pathway. However, we observed that p53 protein expression level was down-regulated at all the concentration of Al(mal)_3_ except 100 µM (Figure 6A). Also p21 protein, which is the downstream effector molecule of p53 pathway, was also reduced at all the concentrations of Al(mal)_3_ (Figure 6A). So, we next analyzed the ubiquitination status of p53 to ascertain whether its levels are being post-translationally suppressed. However, no significant poly-ubiquitination could be observed, and only bands corresponding to mono-ubiquitinated p53 could be seen (Figure 6B). Hence, we studied the transcriptional status of p53 gene and observed decreased levels of p53 mRNA in Al(mal)_3_ treated cells as compared to control (Figure 6D) indicating that p53 is transcriptionally suppressed in response to Al treatment and plays no positive role in mediating Al induced apoptosis of SH-SY5Y human neuroblastoma cells.

### Caspases Inhibition Rescues Cells from Al-induced Apoptosis

**Figure 7:**
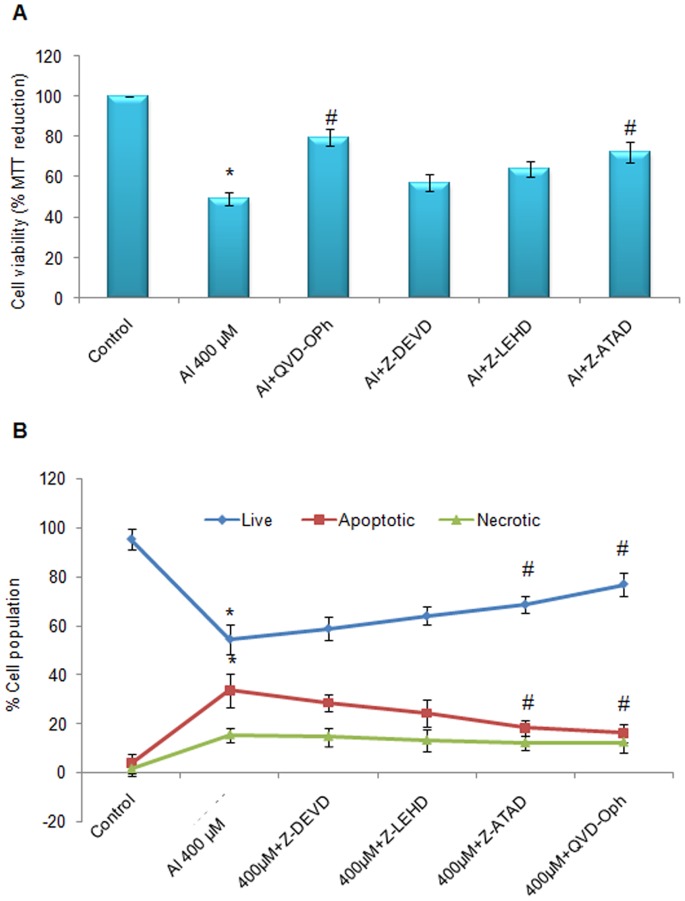
Treatment with pan caspase inhibitor and caspase 12 inhibitor rescues SH-SY5Y cells from Al toxicity. SH-SY5Y cells were incubated with 400 µM Al(mal)_3_ or with 400 µM Al(mal)_3_ and peptide inhibitors of pan caspases (QVD-oph), caspase 12 (Z-ATAD), caspase 9 (Z-LEHD) and caspase 3 (Z-DEVD) for 24 h. Cell viability was determined by MTT reduction (A) and live, apoptotic and necrotic cell populations were assessed by estimation of LDH released (B) as per the protocol mentioned under Materials and Methods. The data are represented as means ±SE of three independent experiments. *P<0.05 vs. control;^ #^P<0.05 vs. Al(mal)_3_ treatment.

Since we observed that caspase 9, caspase 3 (Figure 4) as well as caspase 12 (Figure 5) were involved in promoting apoptosis in SH-SY5Y cells, we sought to determine if inhibition of any of the caspases would rescue SH-SY5Y cells from apoptosis. We determined viability of SH-SY5Y cells challenged with 400 uM concentration of Al(mal)_3_ upon caspase inhibition. Compared to 400 uM concentration of Al(mal)_3,_ maximum protection was accorded by pan-caspase inhibition, which increased cell viability to about 30% as determined by MTT assay (Figure 7A) while decreasing apoptotic mode of cell death by 16% as indicated by LDH assay (Figure 7B). This was followed by ER specific caspase-12 inhibitor which significantly increased cell viability up to 21% measured by MTT assay and decreased apoptosis up to 14% as indicate by LDH assay. Caspase-3 and caspase-9 inhibitors increased cell viability up to 7% and 13% respectively and decreased apoptosis by only 5% and 9% (Figure 7A and 7B). The results reveal the prominent role played by ER specific caspase 12 in apoptotic signalling cascade evoked by Al(mal)_3_ treatment.

## Discussion

The role of environmental factors in ageing, neurodegenerative disorders and neuronal apoptosis is not conclusive. In the present study, we have attempted to investigate the role of Al, which is still controversial with respect to its relation with Alzheimer’s and other neurodegenerative disorders. We have chosen human neuroblastoma SH-SY5Y, which is a relatively homogeneous neuroblast like cell line that has been extensively used as model of neurons since these cells posses many biochemical and functional properties of neurons. SH-SY5Y cell line is being widely used in experimental neurological studies involving functional analysis of neuronal differentiation, metabolism, neurodegenerative and neuro-adaptive processes, neurotoxicity, and neuro-protection [26], [27]. Therefore, SH-SY5Y cell line is a good in-vitro neuronal model system to evaluate toxicity of Al. Our results revealed that Al(mal)_3_ induced cytotoxicity in time and dose dependent manner, which was supported by decreased levels of pAKT protein, an important component of cell survival pathway. From the results of Lactate dehydrogenase assay and Annexin/PI staining, apoptosis was observed to be the main mode of cell death at 200 µM and 400 µM concentrations, which, at higher concentrations, transformed into necrosis (Figure 2C and 2D). Significant activation of caspase 9 and caspase 3 activities further confirm apoptosis as the major mode of cell death at lower concentrations of Al(mal)_3_. Further the role of specific caspases in Al induced cell death was confirmed by the use of peptide inhibitors of caspases. Pan-caspase inhibitor revealed significant protection against Al-induced cell death (Figure 7A). Following pan-caspase, maximum protection was accorded by caspase12 inhibitor (Figure 7A). These results convey that, both endoplasmic reticulum and mitochondria specific caspases are involved in Al-induced cell death and ER specific caspase 12 may play key role in observed cell death.

Apoptosis, while on one hand, is fundamental to normal development and maintenance of tissue homeostasis, on the other, is also a process by which physiologically normal cells may die under neurotoxic conditions [28]. Al has been shown to cause oxidative stress and promote apoptosis through mitochondrial intrinsic pathway [29], yet another study has shown it to be p53 mediated in Neuro2a cells [14]. Our results revealed concentration-dependent increase in cytosolic cytochrome C, while pro-apoptotic protein Bax, which is responsible for disruption of Mitochondrial membrane potential (MMP), increased at 100 µM and 200 µM concentrations but decreased at higher concentrations. The observed effect may be due to necrosis being the main form of cell death at higher concentrations. Bcl2, an anti-apoptotic protein, was down regulated at both mRNA and protein levels as the Al concentration increased. These observations suggest that Al induced perturbation of cellular Bax/Bcl_2_ ratio which promoted the initiation of apoptotic events.

Glutathione (GSH) is an abundant antioxidant in cells that prevents oxidation of cellular macromolecules from the action of reactive oxygen species [30]. In the present study we observed that initially during the first 6 h, as the Al(mal)_3_ concentration increased from 100 µM to 400 µM, the GSH levels also increased, however, at higher concentrations of 500 µM and 600 µM, its levels were considerably low. Nevertheless, at 24 h, the GSH levels were significantly reduced when compared with earlier time periods for all concentrations. The possible reason for the increased levels of GSH at the initial time periods may be in part due to the activation of certain pathway which maintains the intracellular GSH pool. A study by [31] suggested that maintenance of GSH is a novel physiological role of the IKKβ-NFκβ signalling cascade to prevent oxidative damage and preserve the functional integrity of the cells. NFκβ activates in response to oxidative stress insult upon Al-sulphate treatment in human brain cells [32].

The possibility of compromised cellular antioxidant defenses was confirmed by decreased Nrf2 levels, which regulates the protective mechanism within the cells against oxidative stress by the transcription of antioxidants enzymes [33]. A number of studies have revealed that pathological conditions exacerbated due to oxidative burden often involve diminished survival responses [34]–[37]. In addition, a number of neurological insufficiencies have been linked to impaired Nrf2-signaling that enhances the susceptibility to oxidative stress [38]–[40]. We observed significant reduction in protein levels of Nrf2, and its regulated downstream target NQO1, which confirms that Al exposure causes dysregulation of Nrf2 pathway, aggravating free radical generation by compromising cell’s potential to counteract the oxidative stress. Nrf2 stability is a critical determinant of its functional capacity. While Keap1 is said to perform a key role in suppressing cytosolic Nrf2 levels, many other mechanisms co-exist which may influence Nrf2 activity. A recent study has related the weakening of cellular antioxidant defenses to stress-mediated Akt-deactivation leading to compromised Nrf2 stability [41]. The observed down-modulation of Akt phosphorylation due to Al exposure in SH-SY5Y cells may explain for the diminishing Nrf2 and NQO1 levels.

Tumor suppressor gene TP53 activates during genotoxic stress and promotes cell cycle arrest by the activation of p21 [42]. Wild-type p53 is required for the normal function of the p53 protein. It is well reported that SH-SY5Y cells harbor wild-type p53 [43], [44]. Several studies employing single-strand conformational polymorphism analysis to detect p53 mutations have shown that p53 is rarely mutated in neuroblastoma tumors and cell lines [45], [46], [43]. Further, studies have shown intact p53 signaling and activation of p53 in terms of nuclear localization of its protein in response to drug and irradiation treatment in SH-SY5Y cells [47]–[49], [43], [44]. Johnson *et al.* [14] demonstrated increased transcript levels of p53 during Al induced toxicity in Neuro 2a cells, while, on the other hand, a study by Daniela *et al.* [50] demonstrated that oxidative stress mediates DNA damage-induced apoptosis through p53-independent pathway in Alzheimer patient’s fibroblast cells. Our results showed that p53 protein level was up-regulated only at 100 µM concentrations while at rest of the concentrations, as Al induced its cytotoxic effects, p53 level decreased in comparison to control. p21, the downstream molecule of p53 pathway, was also down regulated at all the concentrations of Al(mal)_3_ which suggest the inactivation of p53 pathway. In order to explore the reason for decline in p53 protein, we evaluated p53 ubiquitination. However, no significant poly-ubiquitination could be observed, except for bands corresponding to monoubiquitinated p53. Hence, we next estimated the mRNA status of p53 gene and observed that its transcript was down regulated at all the concentration of Al(mal)_3_. These findings collectively indicate that the observed downregulation of p53 protein in Al(mal)_3_ treated cells may not be post-translationally regulated, rather, transcriptional down-regulation is responsible for the observed effect. Our results show that the apoptotic pathway induced by Al(mal)_3_ in human neuroblastoma cells is p53- independent.

Endoplasmic reticulum stress is implicated in many neurological disorders [51]. CHOP/GADD153, sensor of endoplasmic reticulum stress, is highly up-regulated during ER stress [52]. Increase in intracellular calcium and activated caspase-12 are also markers of ER stress [53]. We observed caspase 12 activation, enhanced intracellular calcium levels together with increased mRNA and protein levels of CHOP/GADD153 that reached their maximum at 400 µM Al(mal)_3_ concentration. Previous studies have reported that ER is also the site of Aβ generation which is a hallmark of Alzheimer’s disease [54], [55]. While, on one hand, reports have shown that Aβ induces Ca^2+^ release from endoplasmic reticulum (ER) stores [56], others report that influx of Ca^2+^ through calcium channels of the plasma membrane or through release from ER stores increases Aβ generation [57] by alteration in the metabolism and production of Aβ [58]. Whatsoever, an intricate relationship between Ca^2+^ dysfunction and Alzheimer’s disease does exist [59]. In our study, we have observed an association between increased intracellular calcium and Aβ(1–40) levels in response to Al(mal)_3_ treatment. Both Aβ(1–40) and Aβ(1–42) have been reported to form oligomers and protofibrils [60]. Our results show that the formation of Aβ(1–40) oligomers is induced in the presence of Al(mal)_3_ and interestingly, as the Al(mal)_3_ concentration increased, the levels of oligomeric Aβ(1–40) were observed to enhance. In particular, at 400 µM concentration, the level of Aβ(1–40) corresponding to oligomeric form was significantly enhanced in comparison to its monomeric form. We contemplate that Al may promote the aggregation of intracellular amyloid beta in response to perturbed calcium levels and induces ER stress resulting into activation of apoptotic pathway governed by ER specific caspase-12.

## Conclusion

**Figure 8:**
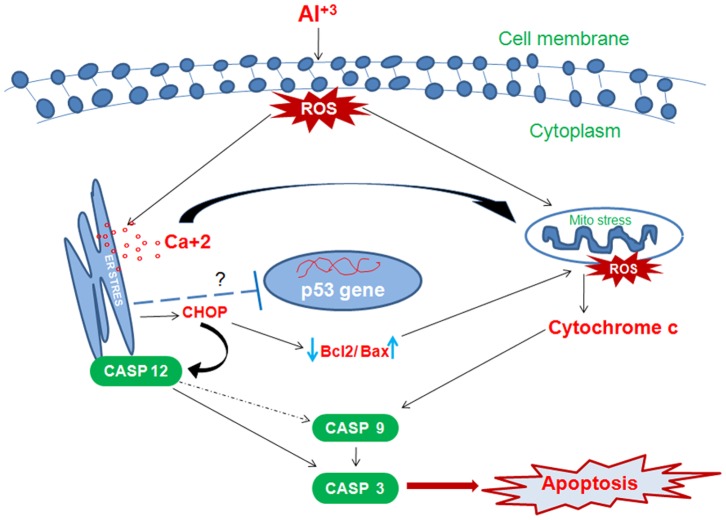
Possible signaling mechanism involved during Al toxicity in human neuroblastoma cells. Al induces oxidative stress which perturbs the endoplasmic reticulum resulting into the release of calcium and ER stress related proteins like CHOP and Caspase 12. The altered cellular calcium levels disturb mitochondrial membrane permeability and induce cytochrome c release which activates caspase cascade. ER specific caspase 12 is also implicated in the activation of apoptosis through direct activation of caspase 3 or by caspase 9. Besides this, CHOP also disturbs the ratio of Bax/Bcl_2_. ER stress may be implicated in the inactivation of p53 in response to Al(mal)_3_ toxicity or other unknown possible mechanism may be responsible for suppressing transcript levels of p53.

In all, the study shows that oxidative stress and consequent apoptotic hallmarks begin to establish within the cells at a concentration as low as 200 µM reaching to their maximum at 400 µM where apoptosis is observed as the main mode of cell death. Further, p53 independent apoptotic pathway entailing largely endoplasmic reticulum stress is implicated in Al(mal)_3_ mediated cell death in human neuroblastoma cells (Figure 8). Increase in intracellular calcium levels and amyloid β explicate the association of Al with ageing disorders like Alzheimer’s that may help to understand the insight of Al induced neurotoxicity and the molecular mechanism involved in neuronal apoptosis.
